# The business of death: a qualitative study of financial concerns of widowed older women

**DOI:** 10.1186/s12905-015-0194-1

**Published:** 2015-04-18

**Authors:** Michelle DiGiacomo, Joanne Lewis, Jane Phillips, Marie Nolan, Patricia M Davidson

**Affiliations:** University of Technology Sydney, Faculty of Health, PO Box 123, Broadway, New South Wales, 2008 Australia; Johns Hopkins University, School of Nursing, North Wolfe Street, Baltimore, MD 21205 USA

## Abstract

**Background:**

The feminisation of ageing and increasing number of widowed women in contemporary society has significant implications. Older women are at risk of poor health, social, and economic outcomes upon widowhood. The aim of the study was to describe women’s experiences in the period soon after their husbands’ death, including their financial issues and concerns, and the ways in which these experiences impacted on the transition to widowhood late in life.

**Methods:**

This was a longitudinal study using serial in-depth semi-structured interviews with 21 community-dwelling women over the age of 65 in Australia. Verbatim transcripts underwent Interpretive Phenomenological Analysis.

**Results:**

Thematic analysis revealed: 1) administrative burden increases vulnerability; 2) gender roles impact on transitions; and 3) financial adjustments render housing insecurity and health risk. High administrative burden within the context of significant grief and mourning was a defining feature of the early bereavement period. Complicated protracted administrative processes, insensitive interactions, and reminders of loss contributed to distress, anxiety and feelings of demoralisation. Several women identified assumption of household financial management as the most difficult aspect of coping with their husband’s death.

**Conclusions:**

Older women may have unmet needs for assistance with administrative, financial, and legal issues immediately following spousal death and potentially for years afterward. Lack of familiarity and absence of instrumental support with financial and legal issues signal the need for policy reform, resources to improve financial literacy in women throughout the life course, increased advocacy, and consideration of different support and service models.

## Background

As in many other developed countries, Australia’s population is aging and this is expected to continue [1]. Despite a projected gradual convergence in life expectancy, women are still living longer than men in Australia. The feminisation of ageing and increasing number of widowed women in contemporary society has significant implications [2,3]. Literature documenting outcomes of spousal bereavement are replete with physical and emotional health sequelae [4]. Research has shown strong associations between women’s biological factors and spousal death which suggests physiological vulnerability to this life event [5]. Health events, described as exacerbations of current conditions, new diagnoses, or incidents requiring health professional intervention, have been reported in older women in the early bereavement period, as have changes in health and risk behaviours [6]. Increased rates of hospitalisation, death, depression, and loneliness have been reported and suggest unmet need for health and social services involvement [4,7].

Social and economic ramifications of widowhood increasingly have been documented [8] as has recognition that this important life event is shaped by social, economic, historical, political, and cultural factors [9]. Economic risks faced by the current population of older women may reflect their longevity coupled with interrupted or lower level employment over a lifetime. Traditional gender socialisation for women born in the 1920s-40s [10] positioned them in the home rearing families (rather than in the workforce earning money and managing household finances, except during war time) and men as financial providers through paid employment [11]. Regardless of socioeconomic status, a lack of experience in managing household finances can challenge the transition to widowhood [12,13].

Death of a husband, or long-term male partner, changes a woman’s living arrangements. In 2011, 32% of women age 65 and over lived alone in Australia and 69% of these women were widowed [14]. Financial need, poor health status, or preference to live with family are among factors that may influence a woman’s decision to cohabitate upon spousal death [15,16]. Others may prefer to live alone and retain their independence, although for many older women, this may be the first time they have ever lived alone [15]. Despite many positive aspects of living alone, negative outcomes can include social isolation, financial strain, and inadequate support to facilitate chronic disease management [15,17]. Loss of spousal income and sustained or increased expenses associated with loss of economies of scale can influence the level of financial stress women experience following their husbands’ death, whereby expenditure exceeds income [18,19]. Although the majority of Australian seniors receive some publicly funded income support based on level of financial need (the Age Pension) [20], transfer from a joint to a single pension reduces this support by 40%, potentially destabilizing standards of living [21].

While much bereavement research has focussed on physical and psychological health outcomes, less attention has been paid to more practical outcomes associated with day-to-day management in the early bereavement period. Despite acute grieving in the days and months following spousal death, necessary administrative tasks loom. Examples of such tasks include notifications of death and provision of copies of the death certificate to various government and non-government organisations where accounts must be closed or transferred. Administrative burden has been defined in industry as the effort required to supply mandatory information required by law and regulations [22]. The complexity of bureaucracies and increasing reliance on the internet for enquires and lodging claims may make it difficult for older women to navigate these financial pathways. Familial and other support networks may provide practical assistance with these tasks initially, but we know little about women’s adjustment to living and managing alone during this early period. The few exceptions to this paucity of research have identified household maintenance [23] and financial insecurity [24,25] as pertinent stressors for older widows. A minority of women are at higher risk of adverse events [6] and inadequate adjustment [23] during this transition, yet few interventions identified in the literature have addressed needs beyond direct physical and psychological health outcomes [3]. By ascertaining ways in which perceived stressors associated with this transition are experienced, we can inform design and delivery of services and supports to circumvent or alleviate such burdens or stressors. Thus, we undertook a study to elicit contemporaneous descriptions of older women’s experiences and needs for information, services, and support upon spousal bereavement.

## Methods

This study was informed by an ecological framework, as well as life course, social role, generational cohort, and stress process theories, which guided data collection and interpretation. The ecological framework asserts that health and wellbeing are affected by a dynamic interplay of biological, behavioural, and environmental factors throughout the life course of individuals, families, and communities [26]. It assumes that age, gender, race, ethnicity, and socioeconomic differences shape the context in which individuals function, thus influencing health risks and resources directly and indirectly. The life course theory appreciates the historical, social, and geographical contexts that shape an individual’s life [27]. Thus, major societal events that occurred during different life stages, particularly childhood, contribute to shaping the individual’s attitudes, beliefs, and behaviours. Social role theory suggests that the sexual division of labour and societal expectations based on stereotypes produce gender roles [28], although traditional gender stereotypes mainly represent the dominant (white, middle-class) culture [29]. One example is the notion of the nuclear family whereby the man (husband) is the provider who earns and manages household finances and the woman (wife) is the nurturer who is responsible for home duties and raising children. Generational cohort theory [27,30] suggests that historical events and social changes, particularly those experienced during formative years, affect the values, attitudes, beliefs, and behaviours of individuals and have lasting consequences. For example, women born in the 1930s, who lived through major events such as World War II and the Great Depression, may have learned to live with less and endure hardship quietly [31,32]. Conservatism and social norms of the 1950s may have influenced a woman’s decision not to join the workforce or engage in household financial management.

The stress process model asserts that contextual factors can have implications for types, appraisals, and outcomes of stressors [33,34]. For example, a woman’s personal resources, such as social support, may influence her appraisal and subsequent response to a given stressor. Thus, we argue that a woman’s experience and negotiation of early widowhood is shaped by her social environment and roles, socio-historical events and policies, and her experiences, appraisals, and resources.

### Recruitment and data collection

Recruitment and data collection took place from August 2009 - November 2011. A convenience sampling approach was adopted whereby study advertisements appeared in health, social service, government and non-government, organisation newsletters and websites targeting health service professionals and older women. Study brochures were made available at hospital palliative care memorial services which enabled potential participants to express interest in the study via telephone, email, or health professional. Participants were included if they were women, aged 65 and older, had experienced the death of their husband/partner within the past two years, and were able to read, speak, and understand English. Recruitment ceased upon data saturation wherein concurrent analysis signalled that no new information was emerging from interviews.

Demographic data were collected following recruitment. Our conceptualisation of adjustment to widowhood as a multifaceted transitional process informed our study design as a series of interviews and surveys over a twelve month period [35]. Each woman participated in three in-depth semi-structured interviews at 6 month intervals with a female interviewer (MD) who was previously unknown to them. Time 1 interviews lasted between one and three hours and were conducted at participants’ homes or via telephone depending on geographic location, availability, and participant preference. The interview schedule reflected aspects of the ecological framework, life course and role theories, and the stress coping process model. The interviewer elicited participants’ descriptions of their experiences leading to and following her husband’s death. Second and third interviews lasted between 20-40 minutes and centred on participants’ experiences since the previous interview, re-visiting some of the issues discussed previously, and clarifying and validating the researcher’s interpretations of previous interview data. The semi-structured format allowed for discussion of emergent topics and clarification of meanings throughout interviews. The researcher conducting and analysing interviews had qualifications in psychology, experience in qualitative research approaches, and working with older women in health settings. All interviews were digitally recorded with permission of participants and transcribed verbatim. Pseudonyms were used to identify participants’ excerpts following de-identification of data.

### Ethical considerations

Conduct of this study was approved by the St. Vincent’s Hospital and Curtin University Human Research Ethics Committees and was conducted in accordance with the National Health and Medical Research Council and Helsinki Declaration [36]. The researcher telephoned participants the day after interviews to assess their mood and link to support as required. Upon conclusion of final interviews, participants who were socially isolated or who had recently experienced a serious health event were followed up regularly by phone.

### Data analysis

All transcripts underwent Interpretive Phenomenological Analysis (IPA) [37] which centres on individuals ascribing meaning to their experiences in their interactions with the environment. As its name suggests, IPA is a set of systematic processes that shift from phenomenological to interpretive while focusing on the participant’s perspective and understandings in different contexts [37]. It is an iterative, inductive, and flexible approach. Analysis involved: 1) close reading and re-reading of transcripts while note-taking in margins [38]; 2) ‘bracketing’ the analyst’s critical perspective; 3) recording critical and interpretive comments in a reflexive diary; 4) re-reading the text and identifying codes and themes that best capture the essential qualities of that interview while also looking for connections between themes; 5) revisiting earlier transcripts to re-consider data; 6) clustering themes and concepts and developing an overall structure using excerpts from interviews; and 7) re-assessing and revising new themes against earlier data [38]. To facilitate rigor, a second researcher independently coded unmarked transcripts [39]. Differences in coding were resolved through discussion. These steps were undertaken for each data collection period. Longitudinal data were managed with matrices to enable comparison across participants, themes, and data collection phases. Findings were discussed with a project advisory committee, which included consumers, for consultation purposes prior to manuscript submission.

## Results

Participants’ demographic characteristics are presented in Table 1. Initially 24 participants were recruited, but 3 later declined before the first interview due to lack of time available (1) and a natural disaster (flood) (2). The 21 participants were an average age of 71 and mainly living alone in houses at the time of the first interview which took place between 2 and 47 months after their husband’s death (median 12 months). The majority of participants (76%) had been caregiving for their ill husbands [6] for three or more months prior to their deaths. Five women experienced their husbands’ deaths as unexpected or within three months of illness onset. Of the caregivers, duration of caring occurred for an average of 3.3 years, yet this ranged from 3 months to 12 years. Participants were aged 63-81 at the time of their husband’s death and had been married an average of 43 years. Mainly, participants had been born in Australia, were living in major cities, and nearly half were living in New South Wales. The majority of participants identified as retired (85%) and almost half of participants had not completed year twelve (47%). One participant died before her second interview.

**Table 1:** **Demographic characteristics of participants (N = 21)**

	**Mean (SD)**	**Range**
**Statistics**		
Age	71.43 (6.13)	63 - 82
Number of children	2.24 (1.58)	0 - 5
Number of years married	43.14 (15.12)	8 - 63
Number of months bereaved	14.29 (11.07)	2 - 47
Caregiving duration	3.3 years	3 months – 12 years
**Country of birth**	**%**	**N**
Australia	76.2	16
England	19	4
USA	4.8	1
**Veteran’s entitlements**	9.5	2
**Living situation**		
Living alone	95.2	20
Living with family	4.8	1
**Type of dwelling (Time 1)**		
House	66.7	14
Unit/townhouse (non-retirement)	19.0	4
Retirement village	14.3	3
**State**		
New South Wales	47.6	10
Queensland	33.3	7
Australian Capital Territory	14.3	3
Western Australia	4.8	1
**Remoteness Area Classification**		
Major Cities	66.7	14
Inner Regional	19.0	4
Outer Regional	14.3	3
**Employment status**		
Retired	85.7	18
Working part-time (paid)	9.5	2
Self-employed	4.8	1
**Highest level of education**		
Completed primary school only	4.8	1
Left high school before intermediate or school certificate	4.8	1
Intermediate or school certificate (Year 10)	38.1	8
Leaving or higher school certificate (Year 11/12)	19.0	4
Apprenticeship/trade qualification	4.8	1
Diploma	4.8	1
University degree	14.3	3
Other	9.5	2

Three participants had contacted the researcher and expressed interest in participating during the recruitment phase, although they exceeded the age and time since death inclusion criteria. Given the exploratory nature of this study and ethical considerations, there was scope to adopt a more inclusive approach to recruitment rather than reject involvement of these three participants. Examination of data revealed no significant differences between these and the other participants.

Results pertaining to physical health and caregiving experiences of the women are described elsewhere [6,31]. Pertinent to women’s experiences of recent bereavement were ways in which administrative and financial burdens and difficult interactions with organisations impacted on wellbeing and adjustment. Themes derived from women’s experiences were: 1) administrative burden increases vulnerability; 2) gender roles impact on transitions; and 3) financial adjustments render housing insecurity and health risk.

Discussion of financial and administrative burden peaked during the first round of interviews, with extensive contextual information provided to describe interactions. Interview excerpts presented here, therefore, are mainly from these initial interviews. Upon successive interviews, participants were asked to provide an update of progress since the previous interview, including any administrative or financial issues previously discussed.

### Administrative burden increases vulnerability

The early period following the husband’s death was highly influenced by administrative activities including funeral planning, notification of the husband’s death to a variety of organizations, account name changes or closures, and estate settlements. These activities consisted of contacting or visiting organisations, requesting, completing, and submitting paperwork and legal documents for the purpose of proving identity or relationship with the deceased to transferring ownership of property and accounts. In most cases, these activities became the responsibility solely of the widow, although sometimes family members or friends were able to provide assistance. As depicted in the following excerpts, the majority of women described these activities as physically and emotionally demanding, particularly within the context of significant grief and loss:

*It’s quite complicated with them because you have to go through the Lands Department and have the whole property just put in the one name before they can issue it to the Council. So that’s going to be a complicated thing and I have to go to the Solicitor for that, so yes, I’ll just have to wait until I feel strong to do that.* (Gloria)

*As I always say, life wasn’t meant to be easy, but I don’t think it was meant to be this hard either. It’s bad enough that he’s passed away but to have to go through all that as well, it’s not good.* (Alice)

*I’ve never appreciated the responsibility of being the executor of the Will and it is very hard to do when you are grieving. To send out all those death certificates to all these people… I would like to have been better prepared, what being the executor meant.* (Rebecca)

These excerpts depict the women’s perceptions of the onerous nature of tasks that required physical and emotional stamina. In some cases, the volume of the workload, the cognitive and physical demands of getting to and from multiple appointments, all within the context of significant grief, contributed to women’s increased psychological distress and difficulties coping. Some women described feeling overwhelmed by these demands which contributed to anxiety:

*…That “will I cope?” feeling. In the first few months, I felt terribly anxious and I think I felt overwhelmed with this to do and that to do and how I was going to get it done.* (Helen)

*I have this incredible mess to sort out without anyone to help me at all (crying).* (Fran)

Although some women described proximal support networks, assistance was often variable and time-limited. Most of the women described working through the administrative and financial matters alone.

### Distressing symbolism

Inconvenience and exhaustion were not the only outcomes of bereavement-related administrative burden. In addition, the repeated act of removing the husband’s name from accounts reinforced the reality of loss:

*You have to get in touch with the banks and the solicitors and the Councils and the electricity people and all those sorts of people, to have it in your name only, and that is very distressing – transferring the name… each place you go to, you have to give them a copy of the death certificate.* (Victoria)

Few women were able to undertake these activities detached from this distressing symbolism. Instead, for several women, these tasks painfully illustrated the erasure of the husband from the woman’s life:

*…having to take his name off certain documents and bank accounts, I’m finding all those things are so painful to do.* (Gloria)

The provision of the husband’s personal information to strangers caused additional distress for some women who perceived this as an invasion of privacy and violation of intimacy:

*One thing that upset me…The amount of detail that goes on the death certificate; I cannot see the point in that. All the details of his illness, this goes off to everywhere…there’s no privacy left for him anymore, he would have hated that. Why does the bank manager need to see his personal intimate health details or to PayPal or Frequent Flyers?…I don’t think it’s right, I don’t suppose they shred it once they’ve recorded the personal details.* (Rebecca)

Details of health and wealth were not perceived as the domain of private industry. This lack of privacy contributed to a feeling of being exposed, vulnerable to breaches of privacy, and as though boundaries were eroding and they were losing control.

The interpersonal interactions between these women and various organisational representatives featured prominently in discussions. Interactions contributed to distress, in some cases impacting on their self-concept, as depicted below:

*They don’t stop to think of the consequences of what they’re doing and saying…They just don’t realise how much grief they put a person through…I think this is one of the most appalling things that the family and I have had to go through (crying). It’s bad enough to lose your partner but to go through all this as well is demoralising and it’s wrong. Nobody makes anything easy.* (Alice)

*This has been a huge shake to my confidence as this has been so demoralising…At this time, you certainly don’t need banks telling you you’re stupid or financial advisers.* (Katherine)

Both of these excerpts used the term ‘demoralising’ to characterise the impact of their interactions with various organisations; a term that implies a loss of confidence and spirit. Katherine’s excerpt depicts the distress that directly resulted from her dealings with her bank regarding a joint account on which her husband was the principal account holder. She explained that the bank had invoked a complicated process, blamed her for delays, and charged her additional fees for necessary changes. Throughout this period, she was supported by a solicitor (lawyer) who was enraged at the bank’s treatment of her, although this mitigated neither her distress nor the prolonged settlement process which extended beyond the study period.

### Gender roles impact on transitions

Women described their experiences assuming a financial management role in the year following their husband’s death. Several women whose husbands had previously managed the household finances, linked this role differentiation to gender roles and discussed the implications upon widowhood. One woman explained how her lack of financial involvement impacted on her transition to widowhood:

*Like most women of my vintage, I didn’t have anything to do with the money. I left it all to my husband…Of course when he died, I had to handle everything… I have found it very difficult. If you want to know the worst part of the year in having to cope without my husband, it has been the money…it’ s not knowing anything about it, understanding how it works, because he had shares, managed funds, all those sort of things…If I could have had more financial help, that would have been good. But I ended up having to go to a financial advisor and he charged an arm and a leg. He charged $6,000 for the plan and he is implementing it as well because I don’t understand any of it at all.* (Rebecca)

These excerpts demonstrate one woman’s struggle in assuming management of her finances. She links her lack of understanding and experience to traditional gender roles of decades past where finance was the domain of men. She ultimately contracted services of a professional advisor at significant cost at a time when her income had decreased.

Not all of the women interviewed were uninvolved with their household financial management. Two women described the benefit of assuming these roles prior to their husbands’ deaths:

*One of the things I was very glad about, when my husband retired and I was still working for a while after that,…so I took over the finances. It was one of the best things I had done. I’ve mentioned it to a few other people and they’ve said, when so and so died, ‘I didn’t know where to start and I didn’t know where to find things.’ I had it all at my fingertips which was something of a relief.* (Helen)

These examples highlight the importance and potential benefits of planning and/or engaging in household financial management prior to being thrust into the role upon bereavement.

### Financial adjustments render housing insecurity and health risk

Of the sixteen long-term carers (husband’s illness lasted ≥3 months), half did not provide any explicit statement of financial difficulty. For other participants, however, the financial impact of spousal loss manifested as disrupted or changed economic resources and living arrangements. Three long-term carers (18.7%) reported serious financial problems (facing loss of assets and cash) that caused financial stress and had potential to impact on housing situations. Personal savings and workplace pension plans were drawn upon to subsidise living expenses and household management during protracted financial resolutions:

*I’ve already spent thousands of my [company pension] to catch up on bills because the estate is not through…I’ll be able to see if I can stay in my current home.* (Pat)

This woman used her workplace pension, an account intended to financially support her retirement years, in an effort to remain in her home in the period following her husband’s death. In addition to grief, she was dealing with housing insecurity and a precarious financial situation. The inability to access information regarding settlement timelines was described as frustrating. The protracted nature of estate resolutions contrasted with expeditious reductions in government age pension support; a program meant to help maintain an adequate standard of living in retirement. Income from a joint age pension was reduced to a single pension three months following her husband’s death, although her expenses did not reduce because there was one less member of the household. Ultimately, she employed services of a financial advisor whose substantial fee consumed a third of her income that year. She was forced to make severe changes to her lifestyle as described by the following statement:

*I’m not used to counting every penny*. (Pat)

Some women considered returning to work to increase income, but pension reduction implications and perceived limited employment prospects due to age stifled this prospect.

Another woman explained that a previously undisclosed financial burden was revealed following her husband’s death. She described her shock upon realising the implications of the significant debt which forced her to sell her family home leaving her in precarious housing. This occurred within 18 months following her husband’s death:

*[I found out about] debt that I didn’t know about above and beyond the mortgage. Now that means I just have to sell. There are no two ways about it. I do have to sell this place which I love dearly, but it would be too hard. I’m pissed off about that. I don’t like being put in that position.* (Emma)

*… but he didn’t tell me… so I got my big shock which buckled me at the knees, one day….What a terrific shock to have to leave the home I loved and end up a rentee. Real learning curve!* (Emma)

Evident in these excerpts is the anxiety, indecision, anger, and loss of control felt upon realisation of having to relinquish her home without planned alternatives. At the time of the first interview, Emma foresaw the need to move from her cherished family home. At the second interview, she had sold her home for significantly less than it was worth due to financial pressures and had just moved into rental accommodation that did not satisfy her needs. She chose to remain in her outer regional community, a decision she explained was essential to her wellbeing, despite being situated two and a half hours drive, on average, from her three children. She described her rental accommodation as having inadequate heating which was particularly problematic given the winter temperature. She was looking forward to moving into a better rental within the next ten days, although this might be delayed for reasons beyond her control. Upon the final interview, she had moved into another rental property; her third home in twelve months. She explained that the repeated upheaval and hectic changes over the previous month, led her to neglect her health and not re-fill a necessary prescription. One evening, she became short of breath, called an ambulance, was admitted to hospital, and diagnosed with heart failure. This series of events illustrated the impact of strain she faced consequent of unforeseen changes following her husband’s death and her related lack of self-care.

Others were forced to re-evaluate their current living arrangements in light of sustained costs and physical capacity to undertake property maintenance. The level of upkeep required for indoor and outdoor maintenance matched neither their physical nor financial capacities as exemplified in the following series of excerpts throughout the twelve month interview period with one woman:

- *➢ …I didn’t know how to turn on the gas cans and it was terribly cold…(Time 1)*
- *➢ Well, first of all I had to buy a new toilet which was leaking and then I had a bad fall and had five stitches…(Time 1)*
- *➢ Then after that I had to buy a new washing machine and a hot water system in the one week…(Time 1)*
- *➢ and I had thirty-five tiles fall off the bathroom wall…I can’t afford to pay everybody.(Time 1)*
- *➢ Having the trees lopped costs $400, so I try to do it myself a little at a time with a long cutter. I can’t do what I used to do in the house, like move furniture, replace light shades on the ceiling, and the garden stuff is too heavy (Time 2)*
- *➢ Then after the holiday, I came home and I couldn’t get in. The deadlock wouldn’t work…(Time 3)* (Debra)

Financial strain and the loss of the husband’s previous home maintenance role led to an increase in women undertaking these tasks themselves, despite risk of injury. Extensive wait lists to access government-funded household maintenance support packages were reported by two women. In light of unmet needs for home maintenance support, some women struggled with the incompatible need and desire to downsize their living arrangements:

*I’m gonna have to move because this place is far too big for me, the garden, the huge pool…I don’t really know where I want to live (voice shaky, near tears)…every time it rains, the pool fills and it is just a constant worry…*(Stella)

## Discussion

This study has described older women’s experiences of administrative burden and financial vulnerability during early widowhood. Administrative burden, elsewhere termed ‘administrivia’ and described as ‘death’s companion’ [40], and a ‘labyrinth’ [41], has not typically featured in bereavement literature, despite its ubiquitous nature upon death. Reports from women in the current study described ways in which institutions affect adjustment with examples of insensitive practices that can exacerbate vulnerability. Further, although some participants had adult children, this did not necessarily translate to prolonged support given geographic restrictions, fractured relationships, employment, and other family obligations.

Recognition of the impact of administrative burden during bereavement was exemplified in recent policy initiatives and programs in the United Kingdom (UK) designed to streamline processes by facilitating cross-government communication [42,43]. ‘*Tell Us Once*’ (TUO) is a national voluntary programme that utilises data sharing to enable a single notification process for changes in circumstances, including death and bereavement [44]. For example, upon registration of the death via the secure online portal or in-person, notifications flow throughout government departments including, but not limited to, those that deal with tax and benefits, pensions and income support, driver licencing, passports, housing services, and electoral roll. Following positive consumer and staff evaluation of the pilot program, several, although not all, areas of the UK adopted this system.

Ensuring confidentiality, privacy, and security of personal information are paramount concerns in design, implementation, and maintenance of centralised web-based information portals. Although privacy impact assessments of the UK’s TUO, have not identified adverse effects [45], a similar ‘one-stop-shop’ portal that links personal information, not including death notifications, across government departments in Australia has not been without security concerns [46]. Although utilising this technology to facilitate communication and reduce administrative burden for the bereaved seems a promising solution, infrastructure to support its development and consultation with consumers is required, as indicated by the UK reports [44].

Our finding that older women faced financial stress and housing unaffordability are consistent with the pension decrease upon transfer from a joint to a single pension [21]. Financial strain was associated with this transition when enacted prior to estate settlement, which extended beyond the three-month grace period wherein payments remain at a couple level. This transition rendered some women cash poor and unsure of their futures. Despite protracted degenerative illnesses, estate planning was not universally enacted prior to death, but was noted as one of several after-death expenses. This left a subset of women facing precarious housing situations. Morris and colleagues [47] argued that older private renters are often at serious risk of homelessness if they experience an unexpected financial crisis or if they are forced to move. Less apparent in the literature are reports of expenditure to procure professional financial management assistance and stress associated with short timeframes to transition financially. From the UK again, a cross-sector initiative has been agreed upon to reduce complexities and delays in probate-related matters between advisors and banks [43]. This estate administration protocol was agreed to by banking, legal, and estate management organisations in the UK, to more efficiently handle savings, credit card, and unsecured loan accounts upon customer death. As in the case of TUO, consideration of similar models tailored to local environments is essential.

Health risks assumed through perceived need to reduce expenditure and insufficient support to maintain dwellings or self-manage conditions was also described. The Australian Housing and Urban Research Institute (AHURI) investigated implications of loss of a partner for older private renters [16]. Difficulty maintaining homes and their contents led AHURI to predict a likely greater dependence of Australia’s increasing older population on housing assistance programs [21,47,48]. Older people’s desire to ‘age in place’ has emphasised the need for more affordable housing options and links to supportive services [49]. Our findings noted long waitlists to access government-funded home care packages to assist with home and garden maintenance services which led some to contract costly private vendors. Evaluation of whether such packages are meeting the needs of older women living alone is warranted.

The impact of living arrangements on post-bereavement distress hampered adjustment in some cases. Some women faced relocation in light of increased maintenance responsibilities, particularly if this was formerly their husbands’ role. Investigation into housing options for older women in Australia is warranted. Relocations enacted upon spousal bereavement may have detrimental impact on social capital, financial security, or other ramifications, and consequently affect health and wellbeing. Associated financial concerns and few perceived affordable housing options reflect the increasing trend of single, older women in Australia becoming vulnerable to housing insecurity and in danger of homelessness [50].

We found that several women experienced distress as a result of assuming financial management roles for the first time in their lives. This underscores the importance of accessible, timely, affordable, and ethical financial management and planning services for older recently bereaved women who may identify with a generation who were not socialised to perform such roles. A National Seniors survey found women scored 15% lower on a financial literacy measure than men [51]. Unfamiliarity with financial and legal issues signal need for resources to improve financial literacy in all women throughout the life course, increased advocacy, and consideration of different support and service models. Having a more active involvement in household financial management prior to or during caregiving phases may lessen vulnerability upon spousal death. Awareness and access to free, confidential, user-friendly financial information services that provide education and information on financial issues is important. Design and delivery of services should reflect the needs and preferences of the target population.

It must also be appreciated that needs of older people in our society are expected to change over time as subsequent birth cohorts age. For example, the ‘Baby Boom’ cohort (born between 1945 – 1964) were socialised to be in the workforce, although some maintained traditional gender roles [52]. Changes in labour force participation, home ownership, debt management, and family structure should be considered for future generations, as should development of increased capacity for financial literacy.

Limitations of this study were mitigated by use of serial interviewing and rapport development through prolonged engagement with participants. This was an exploratory study wherein participants retrospectively reported experiences and as such, may have been impacted by the length of time since bereavement. Our responsibility to link participants with support if distressed may have affected subsequent experiences and outcomes.

## Conclusions

In the early bereavement period, older women can be faced with a variety of external stressors and transitional challenges that can hamper their adjustment and wellbeing. Many of these are financial and administrative. Older widows may be exposed to further emotional and psychological strain while undertaking these administrative and finance-related tasks. Some older women can face financial and housing insecurity that can affect both psychological and physical wellbeing. Findings indicate unmet need for support in undertaking these early bereavement tasks. Various international examplars have utilised cross-sector approaches to address bereavement-associated administrative burdens, but these must be considered in local contexts. The findings of this study, although set in Australia, have implications globally. Relative to many other economies, Australia has a mature social service framework. The challenges posed by the increasing ageing population and feminisation of ageing and bereavement necessitate innovative approaches at individual, community, organisational, and policy levels.
